# Data from salivary gland proteome analysis of female *Aedes aegypti* Linn

**DOI:** 10.1016/j.dib.2017.05.034

**Published:** 2017-05-25

**Authors:** Rakhi Dhawan, Ajeet Kumar Mohanty, Manish Kumar, Gourav Dey, Jayshree Advani, T.S. Keshava Prasad, Ashwani Kumar

**Affiliations:** a Department of Community Medicine, Armed Forces Medical College, Pune 411040, India; b National Institute of Malaria Research, Field Station, Goa 403001, India; c Department of Zoology, Goa University, Taleigao Plateau, Goa 403206, India; d Institute of Bioinformatics, International Technology Park, Bangalore 560066, India; e Manipal University, Madhav Nagar, Manipal, Karnataka 576104, India; f YU-IOB Center for Systems Biology and Molecular Medicine, Yenepoya University, Mangalore 575018, India; g NIMHANS-IOB Proteomics and Bioinformatics Laboratory, Neurobiology Research Centre, National Institute of Mental Health and Neurosciences, Bangalore 560029, India

**Keywords:** Proteomics, Salivary gland, Immunogenic, Vectorborne Diseases, Aedes aegypti, Protein–protein interactions

## Abstract

Salivary gland proteins from female *Aedes aegypti* mosquito were extracted and analyzed on high-resolution mass spectrometry. Proteomic data was analysed using two search algorithms SEQUEST and Mascot, which results in acquisition of 83,836 spectra which were assigned to 5417 peptides belonging to 1208 proteins.These proteins were then assigned molecular functions and further analysis revealed biological processes they are involved in using Gene Ontology annotations. Several immunity related pathways were found to be enriched in salivary gland.The data of this study are also related to the research article “Mosquito-Borne Diseases and Omics: Salivary gland proteome of the female *Aedes aegypti* mosquito” (Dhawan et al., 2017) [1]. These data are deposited in ProteomeXchange in the public dataset PXD002468. In addition,a scientific interpretation of this dataset by Dhawan et al. [1] is available at http://dx.doi.org/10.1089/journal.omi.2016.0160.

**Specifications Table**

**Table:** 

Subject area	Biology
More specific subject area	Mosquito proteomics
Type of data	Mass spectrometry raw files, Excel tables, Graph, Figure
How data was acquired	LTQ-OrbitrapVelos ETD mass spectrometer (Thermo Scientific, Bremen, Germany)
	Proteome Discoverer 1.4 and MASCOT search engine (Matrix Science, London, UK; version 2.2)
	Protein database *Aedes aegypti* (www.VectorBase.org, release date21 Oct 2014)
Data format	Analyzed output data
Experimental factors	Salivary gland tissues were obtained from the female *Aedes aegypti* mosquitoes
Experimental features	Qualitative protein analysis of salivary gland tissue of *Aedes aegypti*
Data source location	Goa and Bangalore, India
Data accessibility	Data are available here and via a web application (ProteomeXchange) Consortium (http://proteomecentral.proteomexchange.org) via the PRIDE partner repository with the dataset identifier PXD002468.

**Value of the data**

• To the best of our knowledge this data set is the largest catalogue so far providing insight into the proteome composition of salivary gland of female *Aedes aegypti.*
• Data provides information on roles identified proteins play in biological and functional categories, protein–protein interactions in metabolic pathways, secretory and immunogenic salivary proteins.Overall it enables better understanding of host-vector interaction and disease transmission.
• The data set is a useful resource of proteins expressed in salivary gland of *Aedes aegypti* female mosquitoes and will aid in biomedical research focused on development of transmission blocking vaccine.

## 1 Data

The core of this dataset is the raw and processed data of the LC-MS/MS analysis of salivary gland proteins of female *Aedes aegypti*. The processed data set contains 83,836 MS/MS spectra, which let led to identification of 5417 peptides and 1208 proteins. All the peptides and proteins identified in this study are listed in Supplementary Tables S1 and S2. Twenty nine proteins were involved in immunity related pathways. Another 15 proteins with signal cleavage site were found to be secretory in nature and thus possibly playing critical roles in blood meal ingestion. To assign molecular functions to these proteins, we used VectorBase resource to assign Gene Ontology (GO) terms [2]. Dhawan et al. [1] performed a scientific interpretation of this dataset with the goal to identify salivary gland proteins that can be the attractive candidates for the transmission blocking vaccines.

## 2 Experimental design

### 2.1 Sample preparation

The freshly emerged five hundred female *Aedes aegypti* mosquitoes from laboratory maintained culture were dissected to obtain the salivary gland. The proteins were extracted using 0.5%SDS solution.The extracted proteins were then quantified according to Bicinchoninic acid assay (Pierce®.Cat#: 23225).

### 2.2 In-gel digestion of proteins

**Fig. 1:**
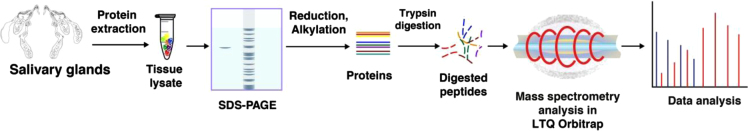
The workflow illustrating the steps involved in proteomic analysis of salivary gland of female *Aedes aegypti*.

200 µg of proteins from salivary gland was resolved on the SDS-PAGE stained with colloidal Coomassie blue. The gel was destained and cut into 18 pieces and subjected to in-gel trypsin digestion as previously described [3]. The destained gel pieces were treated with 0.35 ml of 40 mM ABC and 50% ACN. In-gel proteins were reduced using 5 mM DTT for 60 °C for 10 min and alkylated by adding 20 mM IAA for 10 min in dark. In-gel digestion was carried out using trypsin (modified sequencing grade, Promega, Madison, WI) with enzyme: substrate ratio of 1:20 and incubate at 37 °C for 12–16 h. Peptides were extracted from the gel and dried in speedvac.The workflow illustrating the steps involved in qualitative analysis of salivary gland proteins from female *Aedes aegypti* (Fig. 1).

### 2.3 Mass spectrometry analysis

Mass spectrometry analysis of the 18 in-gel fractions was carried out on LTQ-OrbitrapVelos connected with Proxeon Easy nLC system (Thermo Scientific, Bremen, Germany) as described previously [1]. The LC-MS/MS analysis was carried out in data dependent analysis mode with survey scans (MS) acquired at a resolution of 60,000 at *m/z* 400 and fragment ion scan (MS/MS) acquired at a resolution of 15,000 at *m/z* 400. Both MS and MS/MS scans were acquired in Orbital mass analyzer. Precursor ions were fragmented by high collision induced dissociation (HCD). The parameter settings for LTQ-OrbitrapVelos analysis were a) up to 20 MS/MS scans per duty cycle; b) for MS/MS analysis, monoisotopic precursor mass selection and rejection of singly charged ion criteria were enabled; c) precursor ions were dynamically excluded for a period of 45 s; d) capillary temperature was set at 250 °C; and e) the automatic gain control was set at 1×10^6^ for MS and 1×10^5^ for MS/MS with maximum time for accumulation of 100 for MS and 250 for MS/MS scans. The Polydimethylcyclosiloxane (*m/z*, 445.120025) was used for internal calibration with enabled mass lock option.

## 3 Data analysis

The high-resolution MS/MS spectra were searched against the protein database of *Aedes aegypti* using Sequest and Mascot search engines incorporated with the Proteome Discoverer software suite (Thermo Fischer Scientific, Bremen, Germany). The search parameters used were; a) trypsin as the proteolytic enzyme (with up to one missed cleavage); b) fragment mass error tolerance of 0.1 Da; c) peptide mass error tolerance of 20 ppm; d) oxidation of methionine as a variable modification; e) carbamidomethylation of cysteine as fixed modification. A false discovery rate (FDR) of 1% was applied while identifying the peptide-spectrum matches (PSMs).The bioinformatics analysis of protein data was done using VectorBase resources for assigning Gene Ontology(GO) terms. KEGG pathway portals were used to assess involvement of proteins in different metabolic pathways. Amongst the datasets, immunogenic proteins were identified using ImmunoDB (http://cegg.unige.ch/Insecta/immunodb/), predictive functional analysis of proteins was carried out through NCBI Blastp via blast2go search engine and protein-protein interaction networks were mapped using STRING v 9.1 (Supplementary Table S3) [4], [5].

## Appendix A Supplementary material

Supplementary Tables S1 List of 1208 proteins derived from LC-MS/MS analysis of 18 fractions of *Aedes aegypti* salivary gland proteins.

.

Supplementary Tables S2 List of 5417 peptides derived from LC-MS/MS analysis of 18 fractions of *Aedes aegypti* salivary gland proteins.

.

Supplementary Tables S3 Bioinformatics analysis of salivary gland proteins identified.
